# Magnetic Resonance Imaging of Vermian Lipoma

**Published:** 2014-01

**Authors:** Prashant S. Naphade, Abhishek Keraliya

**Affiliations:** 1 Department of Radiology, Employee’s State Insurance Corporation Hospital, Mumbai, India

## Dear Editor,

**Figure 1:**
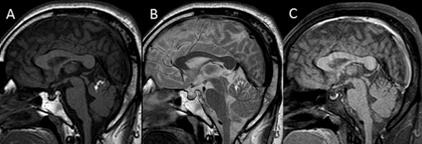
A well-defined, curvilinear T1 (A) and T2 (B) hyperintense lesion is seen in the superior part of the cerebellar vermis. It appears hypointense on T1 fat-saturated images (C), suggestive of lipoma. Superior vermian hypoplasia is also evident.

A 32-year-old man presented with a history of intermittent headaches. On examination, visual acuity was normal and no neurological deficit was seen. Magnetic resonance imaging (MRI) brain scan was performed for further evaluation and revealed a well-defined, curvilinear T1 and T2 hyperintense lesion (measuring 1.2×0.4 cm) in the superior half of the cerebellar vermis. It appeared hypointense on T1 fat-saturated images, suggestive of fat content (figure 1). No evidence of any mass effect or hydrocephalus was seen. These findings were suggestive of vermian lipoma. Superior vermian hypoplasia was also detected, but the corpus callosum was normal. No other abnormality was seen on the MRI brain scan.

Intracranial lipomas represent a congenital malformation with the abnormal differentiation of the meninx primitiva.^1^Most intracranial lipomas are found incidentally, as was the case in our patient. In symptomatic cases, headache and psychomotor retardation are common complaints. Seizures reported in cases of intracranial lipomas appear secondary to the associated anomalies. The pericallosal region as well as the quadrigeminal and suprasellar cisterns is the common location for intracranial lipomas.^2^^,^^3^ Vermian lipomas are rare, with the literature containing only a few such cases.^4^^-^^7^ The morphological variants of intracranial lipomas are the tubulonodular and curvilinear varieties. Intracranial lipomas reveal homogenous fat density (-60 to -120 HU) on plain CT scan and may contain calcific foci within, especially in the tubulonodular variety. Intracranial lipomas display T1 and T2 hyperintense signals with suppression on fat-saturated images. The associated anomalies are better demonstrated on MRI and include dysgenesis of the corpus callosum and vascular anomalies like aneurysm. Surgical excision is not required in most cases.
